# The true cost of cardiovascular imaging: focusing on downstream, indirect, and environmental costs

**DOI:** 10.1186/1476-7120-11-10

**Published:** 2013-04-17

**Authors:** Larissa Braga, Bruna Vinci, Carlo G Leo, Eugenio Picano

**Affiliations:** 1 Institute of Clinical Physiology of Pisa, National Council, Via G. Moruzzi, Pisa, Tuscany, 1 56124, Italy; 2 Research on Research Organization, Duke Medical Center, Durham, NC, USA

## Abstract

To develop a more realistic assessment of costs, herein named “true” costs, the extra-cancer from medical radiation, environmental damage from imaging paraphernalia and radioactive wastes must be included as long-term costs from imaging examinations. It is urgent to define the “true” costs across imaging modalities as it interferes on physicians’ decision to request an exam and on research projects such as cost-effectiveness analysis. Cardiology is the specialty that most will benefit from the outcome as cardiovascular exams represent almost 30% of the total exams acquired annually worldwide.

## From exam total costs to environmental costs and extra-costs from cancer

While innovations in medical imaging represent an exceptional success story, the escalating average costs of imaging is representing a major health economic and societal burden. The cost of diagnostic imaging in the United States (U.S.) is estimated in $100 billion per year [1] and it is steadily increasing. The average total imaging cost per patient per year in the U.S. almost doubled between 1997 and 2006, from $229 to $443 [2]. Several factors contributed to this rapid increase such as high tech modalities, defensive medicine, self-referral, patient demand and overutilization of tests [3].

The estimation of costs across imaging modalities is a crucial information, as it has impact not only in the clinical setting, i.e. physicians’ decision to request an exam; but also research results, i.e. cost-effectiveness analysis. Often, physicians lean toward the imaging modality with the lowest cost for any given comparable accuracy [4]. As only for Europe, if we set the average cost (not charges) of an ultrasound without stress as equal to 1 (cost comparator), the cost of a cardiac computed tomography (CT) is 3.1 ×; single photon emission computed tomography (SPECT) is 3.2 ×; cardiovascular magnetic resonance imaging (MRI) is 5.5 ×; and positron emission tomography (PET) scan is 14.0 × [4]. In addition, the cost range of a certain imaging exam is wide, even between states from the same country. For instance, angiography cost can vary from $1,000 to $4,000; meanwhile, PET exam can cost between $1,100 and $2,700. In our standpoint, so far, we have only imperfect cost evaluation, partial proof of benefit, and incomplete documentation of risk-benefit, typically considered as proven simply by ignoring downstream societal and environmental costs [3,5].

Final or total cost to produce an exam equals the sum of direct and indirect costs. Each institution has its own formula to allocate direct and indirect costs; so total cost variations between institutions is expected. In general, at the imaging department, the direct costs account for the higher percentage of total costs. Direct costs of producing an exam include, but are not limited to, labor, material, film, equipment; while indirect costs include transportation, internet, heating, sewer, lighting, etc.

Herein, we suggest that three factors must be included in the total costs of producing an examination in order to correspond to what we denominate as “true” cost: environmental costs, extra-costs from (fatal and non-fatal) cancer caused by medical radiation, and radionuclide wasting costs. In addition, special attention must be given to the running costs (i.e. electricity) of each imaging modality as it affects greatly the environment.

## A new approach to calculate medical imaging costs

**Figure 1:**
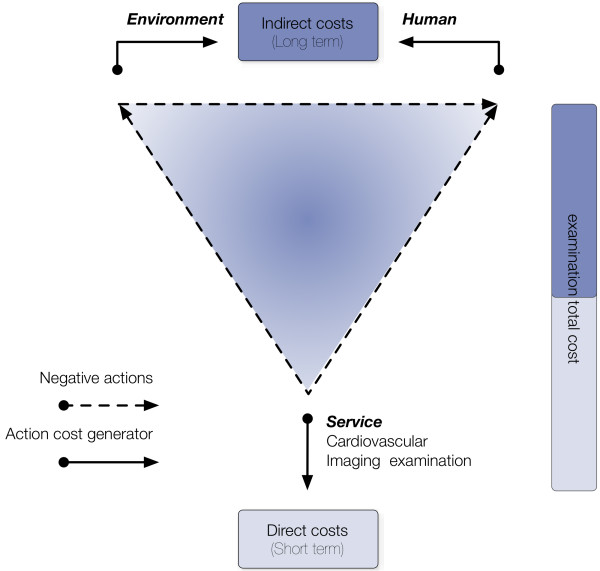
**Illustrates a hypothetical pathway of the “true” costs of imaging including environmental, extra-cancer (human) and radioactive waste costs (service).** Environmental and extra-cancer costs have impact on indirect costs; and radioactive wastes affect both direct and indirect costs, the latter through the environment.

Figure 1 illustrates a hypothetical pathway of the “true” costs of imaging including environmental, extra-cancer and radioactive waste costs. Environmental and extra-cancer costs have impact on indirect costs; meanwhile, radioactive wastes affect both direct and indirect costs, the latter through the environment. As far as the electricity goes, the cost is usually allocated under “institutional cost” having impact only in the direct cost. Herein, we suggest that electricity cost should also be accounted as indirect cost because of its impact in the environment.

## Assessment of environmental costs: still an elusive goal

The long-term costs of environment damage caused by imaging methods paraphernalia have been neglected. In the sustainability era where people are adapting their habits to a more “green” life with the expectation to preserve the ecosystem for future generations, it is more than fair that health care practitioners begin to consider the environment topic in their daily practice. Undoubtedly, the environment cost per imaging methods paraphernalia should be taken into account as part of the “true” costs as its impact not only the ecosystem but also the human health.

The magnitude of the environmental impact, in an economic viewpoint, of certain products and materials during life time can be calculated through life cycle assessment (LCA). Damage to mineral and fossil resources (millijoule surplus energy), damage to ecosystem quality (% plant species * Kilometers squared * year) and damage to human health [disability adjusted life years (DALY)] are the three final categories affected by several factors such as heavy metals, nuclides, hydrochlorofluorocarbon (HCFC), carbon dioxide (CO2), polycyclic aromatic hydrocarbons (PAH), fossil fuels, and land transformation. The same software used to calculate LCA also carry on life cycle costing (LCC) analysis. LCC is a tool that provides a cost estimation of the environmental damage costs and it is expressed through indirect costs.

The environmental cost has impact solely in the indirect costs; however, some of the resources and materials included in the LCA and LCC analyses also affect direct costs (Figure 1). Translating to the imaging setting, for instance, radionuclides and gadolinium are the main components of contrast agents but also materials affecting LCA and LCC results. Contrast is accounted as direct costs; meanwhile, LCC result impacts indirect costs.

Recently, Marwick and Buonocore [6] carried on a LCA to demonstrate the environmental impact of cardiac imaging tests for the diagnosis of coronary artery disease. The study showed that echocardiography is the imaging method that least causes human health harm, ecosystem damage and uses less resources; i.e. the effect of an ultrasound examination on human health was in the order of 1/10 to 1/100th of SPECT and cardiovascular MRI, with 1/5 to 1/50th of the ecosystem effects and 1/20 to 1/100th of their resource utilization. Aside of addressing the environmental damage, the authors briefly recognized the need of evaluating the environmental costs of medical imaging [6].

The inclusion of environmental costs in imaging cost assessment is a conceptual breakthrough: still, some limitations and appropriateness of the employed approach should be considered as a platform for future improvement and refinements of the model.

## Electricity, extra-cancer from ionizing test and radionuclides wastes

The LCA results, and thus LCC, are greatly affected by energy consumption. According to COCIR -on the self-regulatory initiative echo-design of energy using products for medical imaging equipment on December 2011- the energy consumption from imaging methods corresponds up to 80% of environmental impact on LCA [7]. In other words, environmental damage from imaging paraphernalia has positive correlation with scanner size (thus electricity consumption).

Electricity has impact in both direct and indirect costs; the latter through environment (Figure 1). The direct cost of electricity can be calculated by the following formula: kW/hours * electricity price. The electricity price ranges widely depending upon location (country, states and cities). The environment cost, thus indirect costs, can be calculated through LCC, using the CO2 emission as the material (or variable). CO2 emission per day (Kilograms) is equal to consumption of electricity (Kilowatts) times use time (hours) × CO2 emission coefficient (KgCO2/kWh).

The U.S. is the leading country in CO2 emission per capita/year: 20 tons of CO2. The CO2 emission average worldwide is 3.1 tons, and in Italy is 8.1 tons. The cost of 1 ton of CO2 emission is $50. Ultrasound is the modality that emits lowest CO2 per exam (approximately 2.2 Kg in Italy and 2.9 Kg in USA) whereas, 3.0 T MRI emits the highest (229 Kg in Italy and 302 Kg in USA). The cost of CO2 per exam should be also account in the “true” costs.

In the last 10 years, the medical imaging community has become increasingly aware of the need to include long-term cancer risk caused by ionizing radiation in the risk-benefit assessment of ionizing testing [8-10]. This is certainly important for the individual patient and for the society perspective, since small individual risks multiplied by billions examinations become a significant population risks, and up to 10% of all cancers can be due to medical radiation exposure [11,12]. However, this is also a cost and probably a significant one. It has been calculated that only in the U.S., 29,000 new cancers will arise from computed tomography performed in one year [13], and 7,000 new cancers from myocardial perfusion imaging [14].

The costs and savings of technological upgrading in terms of cancer prevention have been addressed by the food drug administration (FDA) some years ago. The group estimated that 723 lives per year spared radiation induced cancer mortality 30 years after the start of implementation of amendments. The average annual financial savings of $519 in the first 10 years of implementation greatly exceeds estimated average annual cost of $49 million to manufactures and to the FDA [15].

Aside electricity and extra-cancer, the cost of radioactive waste also should be accounted in the “true” costs. The costs of radioactive waste have been increasing steadily and it is estimate that in the past 30 years the cost had increased approximately 1000 times: $36/m^3^ in 1980, $14,286/m^3^ in 2005 and $35,714/m^3^ in 2010 [16]. The radioactive waste is accounted as both direct and indirect costs, the latter through the environment.

## Future perspective: addressing the 4 dimension of imaging costs

The assessment of economic, environmental, societal and biologic costs of medical imaging (and its paraphernalia) is becoming increasingly important topic [17,18]. This is especially relevant for cardiologists since cardiovascular imaging represents 29% of the several billion imaging examinations performed annually worldwide [19]. A better, more responsible use of common resources by cardiologists is destined to become one of the new features, and not the least important, of a good practice of medicine.

## Competing interests

The authors declare that they have no competing interests.

## Authors’ contributions

LB and EP designed research; LB conducted literature review, data analysis and manuscript drafting; BV contributed in depth to environmental data analysis and reviewed the manuscript; CGL contributed in depth to economic data analysis and reviewed the manuscript; EP had primary responsibility for final content. All authors read and approved the final manuscript.
